# High‐flow nasal cannula oxygen therapy for respiratory management after postoperative re‐intubation/re‐extubation in patients with trisomy 18 and trisomy 13: Two case reports

**DOI:** 10.1002/ccr3.7090

**Published:** 2023-03-15

**Authors:** Hirofumi Hirano, Yoshie Taniguchi, Masato Kato

**Affiliations:** ^1^ Department of Anesthesiology International University of Health and Welfare Hospital Nasushiobara Japan; ^2^ International University of Health and Welfare Shioya Hospital Otawara Japan

**Keywords:** anesthesia, high‐flow, re‐extubation, re‐intubation, respiratory, trisomy

## Abstract

We present two cases of general anesthesia in children with 18, 13 trisomy. One patient had difficulty with intubation and had to be reintubated postoperatively, another developed postoperative acute respiratory distress syndrome. The use of postoperative high‐flow nasal cannula oxygen therapy to avoid reintubation is considered a feasible strategy.

## Abstract 2

Graphical Abstract

The use of high‐flow nasal cannula oxygen therapy for respiratory disorders in children with severe mental and physical disabilities in the perioperative period is applicable to prevent re‐intubation and additional invasive procedures

## 1 INTRODUCTION

Trisomy 18 and Trisomy 13 are associated with a variety of complications, including severe mental retardation and congenital heart disease, as well as extremely poor prognosis, which has generally precluded aggressive medical intervention in these patients. However, recent reports^1^, ^2^ show that various aggressive medical interventions such as cardiovascular surgery that can improve prognosis have led to increasing opportunities for these patients to undergo general anesthesia and intensive care. We report our experience with high‐flow nasal canula (HFNC) oxygen therapy for respiratory management after postoperative re‐intubation/re‐extubation in two patients with trisomy 18 and trisomy 13.

This article adheres to the Case Report guidelines.

## 2 CASE DESCRIPTION

### 2.1 Case 1

The female patient was aged 2 years and 3 months with a height of 71.8 cm and a weight of 5.35 kg. She had trisomy 18 with mental retardation, interventricular septum defect, interatrial septum defect, patent ductus arteriosus, and pulmonary hypertension. No surgery was performed for these anomalies and only laparoscopic gastrostomy was performed this time.

General anesthesia was induced slowly with sevoflurane and maintained with air, oxygen, sevoflurane, and remifentanil. Anticipating difficulty in intubation due to micrognathia, cervical spinal deformity, limited mandibular elevation, and leftward deviation of the head and neck, instead of trying to intubate with a laryngoscope, we decided to intubate with a video laryngoscope from the beginning. An endotracheal tube with a microcuff diameter of 3.5 mm was inserted into the trachea using a video laryngoscope and a bougie. The field of view with the video laryngoscope was not good. The axis was off and the vocal cords were seen in an oblique position. Intubation was done once, and a leak was noted after intubation. The operation time was 51 min, there was no significant blood loss, and the infusion volume was 150 mL. The patient was extubated in the operating room after the surgery was completed. However, due to markedly labored breathing, she was re‐intubated about 5 min later and admitted to the intensive care unit (ICU).

A planned extubation was performed in the operating room on postoperative day (POD) 5. Hydrocortisone (4 mg/kg three times a day) was administered in advance. The patient remained on continuous intravenous fentanyl at 0.3 μg/kg/h until 2 h before ICU discharge, and then was transferred to the operating room while being administered dexmedetomidine at about 0.3 μg/kg/h.

She was extubated with the bougie left in the trachea in anticipation of re‐intubation. Immediately after extubation, HFNC (MR290, Optiflow Junior; Fisher & Paykel Healthcare, Auckland, New Zealand) was started at a fraction of inspired oxygen (FIO2) of 0.9 and flow rate of 6 L/min. After the patient was observed in the operating room for about 75 min, the bougie was removed, FIO2 was lowered to 0.70, and the patient was returned to the ICU. Blood gas tests were not performed, and the HFNC settings were evaluated and managed by arterial oxygen saturation.

She was discharged from the ICU on POD 6, continued on HFNC (FIO2: 0.2 L, flow rate: 3 L/min) in the general ward until POD 8, and was discharged from the hospital on POD 16 with no further issues or complications.

### 2.2 Case 2

The female patient was 18 years old with a height of 154 cm and a weight of 29 kg. She had trisomy 13 with severe mental retardation and epilepsy. Other medical history or medical concerns included dextrocardia, cleft palate, scoliosis, hearing loss, and left microphthalmia. The patient underwent laparoscopic gastrostomy for difficulty in oral intake and dysphagia.

General anesthesia was induced rapidly with propofol, fentanyl, and rocuronium and was maintained with air, oxygen, sevoflurane, and remifentanil. No problem occurred during surgery or immediately after extubation.

On POD 2, the patient's respiratory condition worsened, and laboratory and abdominal computed tomography (CT) findings were suggestive of acute pancreatitis. She was admitted to the ICU, intubated and ventilated, and subsequently presented with symptoms of acute respiratory distress syndrome.

On POD 14, the patient was extubated with improvement in the findings of pancreatitis and respiratory function. Sedation during ventilation had been maintained with continuous intravenous propofol alone for 2 days, with a dose of 0.5 mg/kg/h at the time of extubation. Immediately before extubation, blood gas analysis showed a partial pressure of arterial oxygen (PaO2) of 83.2 mmHg and arterial oxygen saturation (SaO2) of 96.9% under spontaneous breathing (FIO2: 0.3, positive end‐expiratory pressure: 6 mmHg, pressure support: 6 mmHg).

After extubation, respiratory support with HFNC was started at a FIO2 of 0.40 and flow rate of 50 L/min. Three hours after the start of HFNC, blood gas analysis showed PaO2 77.0 mmHg and SaO2 95.2%. FIO2 and flow rate were tapered according to blood gas analysis results. Subsequently, the patient's body movements became more vigorous, and the day after extubation, she was switched from HFNC (FIO2: 0.35, flow rate: 20 L/min; blood gas analysis: PaO2 64.1 mmHg, SaO2: 92.8%) to nasal cannula oxygen therapy (flow rate: 4 L/min).

She was discharged from the ICU without problems with oxygenation or respiratory status.

## 3 DISCUSSION

The commonality in the two cases presented here is that both patients received active intervention with gastrostomy, but were then re‐intubated, admitted to the ICU, and placed under respiratory management with HFNC after extubation.

Case 1 was a difficult‐to‐intubate case which, despite sufficient preparation and care taken during anesthesia induction and intubation, required re‐intubation probably due to residual expiratory sevoflurane concentration during extubation, airway narrowing due to extubation, and glottis and laryngeal edema despite one‐try intubation and a short operative time. Colleti et al. reported a case of a patient intubated with acute laryngitis in which the use of HFNC after planned extubation avoided re‐intubation.^3^ Thus, planned extubation and subsequent HFNC use may be one way to prevent re‐intubation.

Respiratory problems are common in children, especially those with severe mental and physical disabilities. These patients should be observed with extreme caution immediately after awakening from general anesthesia and extubation, and after re‐intubation. To avoid the need for additional invasive procedures such as tracheostomy triggered by general anesthesia or surgery, careful consideration must be given to respiratory management after extubation and, if re‐intubation results, to respiratory management after re‐extubation.

In case 1, the patient was re‐intubated postoperatively, but the use of HFNC immediately after extubation yielded good results. Based on this experience, in case 2, in which postoperative acute respiratory distress syndrome occurred resulting in re‐intubation, we decided to use HFNC after weaning from mechanical ventilation.

HFNC requires essentially no sedatives and can be readily used in children with severe mental and physical disabilities, who are prone to tongue swallowing and respiratory depression even with the slightest use of sedatives. In case 2, although the patient expressed refusal of HFNC during its use and had to be weaned in the middle of tapering the dose of oxygen, the use of sedatives could have been considered to continue HFNC. The use of HFNC with sedatives has been reported in both children and adults and is particularly common in dental treatment of children with severe mental and physical disabilities.^4^, ^5^, ^6^, ^7^

Reported cases of HFNC use include patients with respiratory disorder or exacerbation of bronchitis or pneumonia due to chronic disease,^8^ airway narrowing after surgery for laryngeal granuloma,^9^ postoperative management of congenital heart disease,^10^ and use for low‐invasive procedures and tests performed under sedation.^4^, ^5^, ^6^, ^11^ HFNC is indicated for a wide range of respiratory disorders, and based on these reports, it may be useful in the perioperative respiratory management of children, especially those with severe mental and physical disabilities.

These patients with trisomy 13 and trisomy 18 who were re‐intubated postoperatively, the use of HFNC after extubation enabled maintenance of a good respiratory status. The results of the present two cases suggest that the use of HFNC for respiratory disorders in children in the perioperative period and children with severe mental and physical disabilities, taking advantage of its features, is applicable to a wide range of cases as one strategy to prevent re‐intubation.

In conclusion, the results of the present case series suggest that the use of HFNC for respiratory disorders in children in the perioperative period and children with severe mental and physical disabilities, taking advantage of its features, is applicable to a wide range of cases as one strategy to prevent re‐intubation and additional invasive procedures.

## AUTHOR CONTRIBUTIONS

**Hirofumi Hirano:** Writing – original draft. **Yoshie Taniguchi:** Writing – review and editing. **Masato Kato:** Writing – review and editing.

## FUNDING INFORMATION

None.

## CONFLICT OF INTEREST STATEMENT

The authors have no conflict of interest to declare.

## ETHICAL APPROVAL

Ethical approval was not obtained because our institution does not require this for case reports.

## PATIENT CONSENT

Our institution requires the consent of the patient for case reports. Written informed consent was obtained from a person with parental authority to publish this report in accordance with the journal's patient consent policy.

Written informed consent was obtained from guardians of the patients to be used for presentation and publication in a journal.

## Data Availability

The data that support the findings of this study are available from the corresponding author upon reasonable request.
